# The gastrointestinal tract and Parkinson’s disease

**DOI:** 10.3389/fcimb.2023.1158986

**Published:** 2024-01-15

**Authors:** Alissa S. Higinbotham, Camilla W. Kilbane

**Affiliations:** ^1^Parkinson's disease and Movement Disorders Center, University Hospitals Cleveland Medical Center, Cleveland, OH, United States; ^2^Department of Neurology, Case Western Reserve University School of Medicine, Cleveland, OH, United States

**Keywords:** Parkinson’s disease, Gut microbiota & dysbiosis, Autonomic nervous system, Prion hypothesis, Mediterranean & MIND diets, GALT (gut associated lymphoid tissue), Vagus nerve, Braak hypothesis

## 1 Introduction

Parkinson’s disease (PD) is the second most common (De Lau and Breteler, 2006) and the most rapidly rising neurodegenerative disease in the world (GBD 2016 Parkinson’s disease Collaborators, 2018); the population of patients living with PD is expected to double over the next twenty years (Dorsey et al., 2018). The rising incidence and prevalence of PD is not fully understood, but factors such as prolonged survival, reduced rates of smoking, and exposures to environmental pollutants and toxins likely contribute (Bloem et al., 2021).

The pathological hallmark of idiopathic PD is the Lewy body, a neuronal inclusion largely composed of aggregated alpha synuclein in the substantia nigra pars compacta dopaminergic neurons and other brain regions (Koga et al., 2021). Clinically, PD is characterized by bradykinesia plus resting tremor, rigidity, and/or postural instability according to the United Kingdom Parkinson’s Disease Society Brain Bank criteria (Gibb and Lees, 1988). PD is preceded by a prodromal period that is often associated with the development of “non-motor symptoms” (**Table 1**). Non-motor symptoms include hyposmia, sleep disorders, such as rapid eye movement (REM) sleep behavior disorder, mood changes, autonomic nervous system involvement, pain syndromes, and cognitive changes (Kalia and Lang, 2015). While both motor and non-motor symptoms contribute to disability, the negative impact on perceived quality of life was found to be larger for the non-motor symptoms of PD (Santos-Garcia and de la Fuente-Fernandez, 2013).

**Table 1:** Explanation of movement disorder-specific terminology used in this article.

Term	Definition	Examples
Motor Symptoms	The classic symptoms of PD that relate to a lack of dopaminergic input into the basal ganglia circuits for movement.	Tremor Rigidity Bradykinesia Postural instability/gait changes
Non-Motor Symptoms	The prodromal and life-long symptoms of PD that are related to PD pathology in peripheral tissues and extra- nigrostriatal brain regions.	Constipation Orthostatic hypotension Urinary changes Hyposmia REM sleep behavior disorder Anxiety Depression Psychosis Cognitive changes Pain syndromes
"Off" Time	Length of time per day that the patient feels his PD medications are not working and he is experiencing troublesome motor or non- motor symptoms.	Upon awakening, a patient may report feeling "bad, shaking, or slow" until he takes the first dose of medication.
"On" Time	Length of time per day that the patient feels her PD medications are working well	Patient may report that each dose of levodopa gives her about 4-5 hours of good symptom control.
"Delayed On" Time	Increased length of time for a given dose of PD medication to take effect or kick in	A patient who takes levodopa four times a day may report the last 3 doses kick in within the usual 20 minutes but that the first dose of the day can take 2 hours to kick in. In this case, the first dose is taking 1 hour and 40 minutes longer than usual to take effect which would be considered "delayed on" time.
Motor Fluctuations	Noticeable periods of "on" versus "off" time related to medication dosing	Early in the course of PD, medications like levodopa can work well and patients feel good as long as they are taking their medication. As the disease progresses, the duration of effect of medication wanes and they have more noticeable "on" versus "of" time, or motor fluctuations
Dyskinesias	Excessive, unwanted, involuntary, random movements related to PD medication kicking in, peaking in effect, and/or wearing off.	Chorea, dystonia, akathisia related to PD medication dosing. Patients may describe dyskinesias as jerking, wiggling, twitching, cramping, or spasm.
Unified Parkinson's Disease Rating Scale (UPDRS)	This is a four-part scale that assesses: Part I: Subjective non-motor symptoms Part II: Subjective motor symptoms Part III: Objective motor symptoms with examination Part IV: Motor complications For each part, higher scores indicate worse disease	UPDRS part III (UPDRS-III) focuses on the motor progression and severity of Parkinson's disease with examination of bradykinesia, rigidity, tremor, and balance in the clinic
Movement Disorder Society sponsored revision of the Unified Parkinson's Disease Rating Scale (MDS-UPDRS)	Revision of the UPDRS by the Movement Disorder Society to better address non-motor symptoms and more mild motor symptoms of early disease	MDS-UPDRS Part IV focuses on the patient's experience with motor complications such as dyskinesias and motor fluctuations

One category of non-motor symptoms involves the gastrointestinal (GI) tract. Sixty-one percent of patients with PD report gastrointestinal-related symptoms (Barone et al., 2009) such as sialorrhea, dysphagia, nausea, vomiting, early-satiety, constipation, bloating, gastroparesis, and colonic dysmotility (Su et al., 2017; Warnecke et al., 2022). These gastrointestinal non-motor symptoms along with aggregated alpha synuclein pathology in the GI tract, can occur a decade or more before the onset of the motor symptoms in the prodromal period of PD (Killinger and Labrie, 2019). Additionally, gastrointestinal issues can negatively impact the absorption of antiparkinsonian medications (Pierantozzi et al., 2006; Fasano et al., 2015; Van Kessel et al., 2019), and motor symptom control (Pierantozzi et al., 2006; Baizabal-Carvallo et al., 2021). Thus, there has been increasing interest in the role of the gastrointestinal tract in the etiology, progression, and symptom severity of both the motor and the non-motor symptoms of PD.

**Figure 1:**
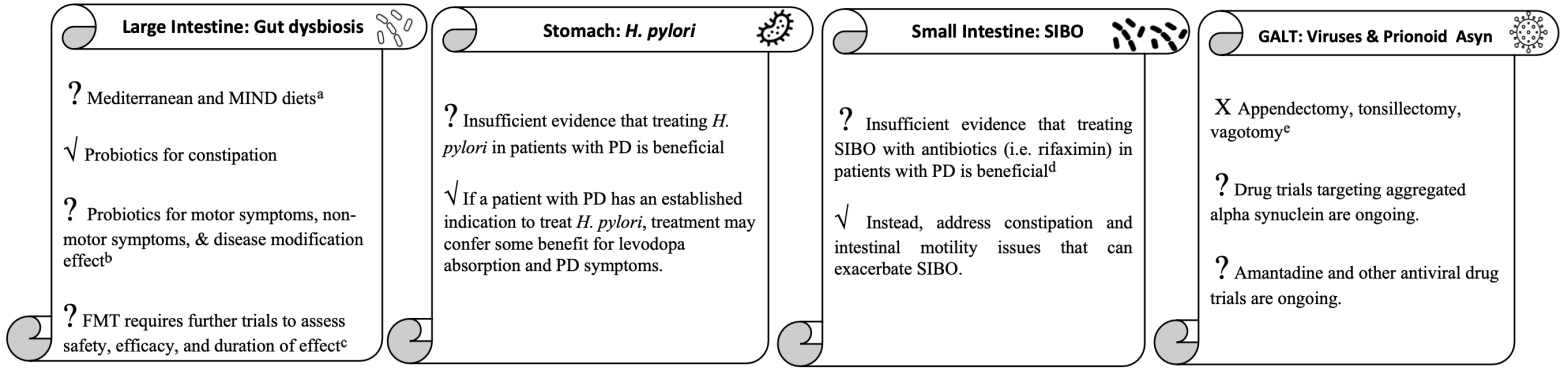
The gastrointestinal tract tour take home points. ^a^More studies needed to clarify effect on patients with existing PD. ^b^More clinical trials needed to determine strain, dose, duration and effect on symptoms other than constipation for which probiotics are currently indicated. ^c^Investigatory for medication-refractory motor fluctuations, dyskinesias, and other symptoms. ^d^Investigatory for medication-refractory motor fluctuations and dyskinesias. ^e^Unlikely to be effective for PD and could worsen inflammation in the GI tract.

To review the role of the gastrointestinal tract in PD in a digestible manner, we will take a tour through the GI tract starting with discussion of the gut microbiota in the large intestine, followed by *Helicobacter pylori* in the stomach, small intestinal bacterial overgrowth in the small bowel, and ending with viruses and prion-like alpha synuclein in the gut-associated lymphoid tissue (**Figure 1**).

We will then address two aspects of the so-called, gut-brain axis: a direct connection between the gut and brain via the vagus nerve, and an indirect connection between the gut and brain via the immune system (Tan et al., 2022). These direct and indirect connections will be relevant to our discussion of alpha synuclein aggregation and propagation, and neuroinflammation, two of the many pathways suspected to play a role in the pathogenesis of PD. Although the etiology of PD is unknown, these and other mechanisms such as oxidative stress, mitochondrial dysfunction, ubiquitin-proteasomal dysfunction, autophagy-lysosomal dysfunction, and axonal signaling and transport dysfunction may play a role (Jankovic and Tan, 2020; Bloem et al., 2021).

## 2 The gut microbiota

The first stop on the gastrointestinal tract tour is the large intestine where the gut microbiota largely resides. The gut microbiota is mostly composed of bacteria which are relevant to PD as they play a role in synthesis and release of neurotransmitters such as dopamine, metabolize drugs such as levodopa, and metabolize nutrients into important signaling molecules such as short chain fatty acids (SCFAs), which regulate the gut mucosal barrier and immune function (Thursby and Jurge, 2017; Metta et al., 2022). The latter role of the gut microbiota protects the body and brain from pathogens, toxins, and inflammation. However, pathogens, toxins, and numerous environmental exposures such as diet and medications can overwhelm the gut microbiota, resulting in a derangement of composition and function known as gut dysbiosis (Thursby and Jurge, 2017).

Patients with PD suffer from gut dysbiosis. Several cross-sectional human studies nicely summarized by Cryan and colleagues examined bacterial DNA profiles from fecal samples and found significant increases and decreases in various bacterial species in patients with PD compared to controls (Cryan et al., 2020). However, there was not much overlap in the profile of bacteria between these studies, presumably secondary to small sample sizes and multiple confounding factors such as age, medications, diet, exercise level, etc. Thus, Hill-Burns and colleagues utilized a larger sample size of over three hundred patients and aimed to understand the significance of potential confounders such as what PD medications the patients were taking. The authors found significant differences in gut microbiota in patients taking catechol-O-methyl transferase (COMT) inhibitors and anticholinergics as well as a borderline signal for carbidopa-levodopa. After controlling for medications and nineteen other important confounders, the resultant bacterial profile yielded some overlap with prior literature including reduced levels of *Lachnospiraceae*, one of the bacteria capable of producing SCFAs (Hill-Burns et al., 2017).

Subsequently, a meta-analysis by Shen and colleagues of fourteen studies aimed to better define the bacterial profile of PD and found significantly lower *Lachnospiraceae, Prevotellacea* and *Faecalibacterium* all of which produce anti-inflammatory SCFAs, and significantly higher *Bifidobacteriaceae, Ruminococcaceae, Verrucomicrobiaceae*, and *Christensenellaceae* in patients with PD (Shen et al., 2021). The former two bacteria may be good bacteria and may reflect a compensatory attempt to combat gut dysbiosis. The latter two bacteria are pro-inflammatory and correlate with more severe non-motor symptoms, respectively (Shen et al., 2021). Reduced *Lachnospiraceae* has also been associated with worse non-motor symptoms such as cognition as well as worse motor scores on the Unified Parkinson’s Disease Rating Scale part three (UPDRS-III) (Barichella et al., 2019).

Finally, most recently, a systemic review and pooled analysis aimed to reduce bias by harmonizing workflow of all studies included and found fourteen bacteria to be differently abundant in patients with PD versus controls (Kleine Bardenhorst et al., 2023). Like the aforementioned 2021 meta-analysis, Klein Bardenhorst and colleagues highlighted the potential importance of reduced SCFA-producing *Faecalibacterium* and *Lachnospiraceae* (Shen et al., 2021; Kleine Bardenhorst et al., 2023). However, these authors also highlighted the potential importance of increased *Akkermansia*, a genus of the family *Verrucomicrobiaceae* that may pathologically degrade the protective mucus layer of the gut (Kleine Bardenhorst et al., 2023). The combination of reduced maintenance of the mucosal barrier by SCFA-producing bacteria and increased mucosal breakdown by *Akkermansia* could render the enteric nervous system and central nervous system more susceptible to pathogens (Kleine Bardenhorst et al., 2023).

While further studies are needed to clarify the hallmark microbiota of PD, it does appear that gut dysbiosis is a phenomenon in PD. Whether such gut dysbiosis is a consequence of PD or a contributing factor to it is an important consideration, however. An animal model of PD study suggests gut dysbiosis can influence symptoms and disease progression (Sampson et al., 2016). The authors took alpha synuclein overexpressing (ASO) mice, which performed worse on motor tasks and had decreased fecal output and water content (a surrogate for constipation) compared to wild-type (WT) mice and made both populations of mice germ free (GF-ASO mice and GF-WT mice). After becoming germ-free, the motor performance of the GF-ASO mice improved significantly, fecal output and water content improved, and motor progression was delayed compared to the ASO mice. Pathologically, the GF-ASO mice had reduced alpha synuclein aggregation in the substantia nigra, caudate, putamen, and frontal lobes and decreased proinflammatory cytokines and activated microglia in those regions as well (Sampson et al., 2016). Other animal models of PD have also demonstrated the potential influence of gut microbiota on motor (Sun et al., 2018; Castelli et al., 2020; Bhattarai et al., 2021; Zhao et al., 2021) and non-motor symptoms (Xie and Prasad, 2020; Zhao et al., 2021). In humans, several strategies discussed shortly have been used to target gut microbiota in PD.

### 2.1 Probiotics

Probiotics have been studied to address gut dysbiosis in PD. Tan et al. randomized seventy-two patients with PD to placebo or a multi-strain probiotic, and after four weeks, patients taking the probiotic had increased number of bowel movements per week and increased quality of life (Tan et al., 2021). Tamtaji et al. randomized sixty-nine patients with PD to a multi-strain probiotic or placebo, and after twelve weeks, patients taking the probiotic had lower inflammatory markers such as c reactive protein (CRP) and better metabolic profiles such as lower insulin resistance. Patients also had lower (improved) Movement Disorder Society sponsored revision of the Unified Parkinson’s Disease Rating Scale (MDS UPDRS) total scores (**Table 1**). The patients continued their PD medication regimen while on the probiotic; however, and given probiotics have been shown to reduce constipation (Tan et al., 2021), it is possible that some of the improvement in MDS-UPDRS scores could be attributed to better absorption of PD medication. Indeed, lack of levodopa pharmacokinetic measurements was a limitation of this study (Tamtaji et al., 2019). More recently, a meta-analysis by Hong et al. including the latter two double blind, randomized, placebo-controlled trials and six other trials revealed a significant increase in the number of weekly bowel movements after probiotics. The trials in this meta-analysis were small with sample sizes ranging from twenty-five to one hundred twenty patients, the duration of treatment varied ranging four to twelve weeks, and the bacterial doses and strain types varied as well, although all eight studies included *Lactobacillus* strain in the administered probiotic (Hong et al., 2022).

Overall, probiotics are an acceptable treatment for the non-motor symptom of constipation in PD (Seppi et al., 2019), and by improving slow transit constipation probiotics may promote more stable absorption of PD medication and thus better motor symptom control (Leta et al., 2023), but more studies are needed to clarify the effect of probiotics on motor symptoms. Additionally, it is plausible that probiotics could influence PD progression given the reported influence of the gut microbiota on various pathways that are potentially implicated in the pathogenesis of PD including inflammation (Tamtaji et al., 2019; Castelli et al., 2020) and alpha synuclein aggregation (Chen et al., 2016), but more work in this field is needed. Future studies will also need to determine the strain, dose, and duration of probiotic required for effective treatment. Furthermore, the probiotic formulation may need to be tailored to the individual patient depending on their medication regimen, age, diet, and personal microbiota, and thus validated, commercially available stool tests would need to be developed (Mirzaei et al., 2022).

### 2.2 Fecal microbiota transplant

Another potential treatment approach to address gut dysbiosis is fecal microbiota transplant (FMT). FMT involves transplantation of healthy, diverse microbiota that are working well in a donor into the gut of a patient suffering from gut dysbiosis (Carlucci et al., 2016). FMT is an effective, FDA-approved treatment to prevent recurrent *Clostridium difficile* colitis (Feuerstadt et al., 2022; Khanna et al., 2022), and interest in the potential of this therapy has expanded beyond gastrointestinal disorders to the field of neurology including Parkinson’s disease (Matheson and Holsinger, 2023).

In an animal model of PD, mice given oral rotenone to induce gut dysbiosis and parkinsonism had improved motor performance and fecal pellet number and water content (surrogate for constipation) after FMT. Inflammation and loss of dopaminergic neurons in the substantia nigra of the rotenone mice was reversed after FMT (Zhao et al., 2021). Similar results were seen after FMT in a 1-methyl-4-phenyl1,2,36-tetrahydropyridine (MPTP) mouse model as well (Sun et al., 2018).

In humans there are limited studies on the efficacy of FMT for PD. The first case report of FMT for PD was in a patient with refractory constipation and tremor (Huang et al., 2019). One week after FMT the tremor disappeared, and UPDRS-III dropped fourteen points. However, the results were not sustained as the UPDRS-III trended back up by three months and the tremor recurred, albeit with less severity. The positive effect on constipation, however, was sustained at three-month follow-up (Huang et al., 2019). In a case series, six patients with PD and constipation were treated with FMT during their routine colonoscopy. Six months after FMT five out of six patients had improved UPDRS-III scores (three of which were deemed to have a clinically meaningful reduction of 3.3 points), and one patient had clinically meaningful worsening of 4.6 points (Segal et al., 2021). Five out of six patients also had improvement on the non-motor symptoms scale (NMSS). Four of six patients had improved constipation by six months, and all reported softer stool (Segal et al., 2021). A prospective study of eleven patients with PD who underwent FMT showed significantly decreased UPDRS-III, Non-Motor Symptoms Scale (NMSS) and Wexner constipation scores at six- and twelve-months post-transplant compared to baseline (Kuai et al., 2021). In a randomized controlled trial of twelve patients with PD, FMT administered orally via capsule for twelve weeks resulted in significantly increased SCFA-producing bacterial strains, decreased constipation and improved small bowel, colonic, and whole-gut transit times, but only transiently improved motor symptoms (DuPont et al., 2023). Finally, and most recently a randomized, double blind, placebo-controlled trial of single dose FMT via colonoscopy in forty-four patients with PD showed no significant difference in MDS-UPDRS parts I-III at six months. However, the FMT group exhibited lower Beck Anxiety Inventory scores and required less levodopa daily than the placebo group (Scheperjans et al., 2023).

FMT is an interesting potential approach to gut dysbiosis in PD, but it has some challenges. There is question of the safest and most effective method of delivery: colonoscopy, nasogastric tube, or oral or rectal capsule (Wang et al., 2016), there is question of duration of effect and need for repeat procedure with time (Huang et al., 2019), and there is concern for transplantation of unwanted microbes (DeFilipp et al., 2019). In terms of safety and side effects, in a systematic review of over one thousand patients with refractory *Clostridium difficile* colitis, inflammatory bowel disease, antibiotic-associated diarrhea, and inflammatory bowel syndrome, significant adverse events, including death and infection, were seen in nine percent of patients undergoing FMT (Wang et al., 2016). In the limited aforementioned FMT studies on PD patients specifically, the most common side effects at follow-up were abdominal pain and flatulence in four of eleven patients and need for ventilatory support in two of eleven patients that subsequently resolved. No other serious adverse events were reported (Kuai et al., 2021). In the case series of six PD pts who underwent FMT, one patient had episodes of vasovagal pre-syncope within a day of the FMT that resolved after eight hours, and no other adverse events were reported (Segal et al., 2021). The two randomized controlled trials discussed reported that the procedure was overall safe with only mild to moderate transient gastrointestinal side effects (DuPont et al., 2023; Scheperjans et al., 2023). Overall, further studies with larger sample sizes are needed to better understand the safety and efficacy of FMT in the PD population.

### 2.3 Antibiotics and proton pump inhibitors

An alternative way to address gut dysbiosis, rather than adding good bacteria with probiotics or FMT, is to eliminate harmful bacteria with antibiotics. This takes us to the next stop on the gastrointestinal tract tour, the stomach, where *Helicobacter pylori* (*H. pylori*), a bacterium classically known to increase the risk of peptic ulcer disease and gastric cancers, resides (Santos et al., 2020). In addition to gastrointestinal diseases, *H. pylori* is implicated in neurological diseases such as PD given its reported ability to increase inflammation, one of the potential pathogenic mechanisms of PD, (Alvarez-Arellano and Maldonado-Bernal, 2014; Shamsdin et al., 2022) and affect levodopa absorption (Pierantozzi et al., 2006; Zhong et al., 2022).

A recent meta-analysis found increased (worsening) in UPDRS-III scores, higher levodopa doses needed for symptomatic treatment effect, longer latency period for levodopa to take effect, and shorter duration of levodopa effect in PD patients infected with *H. pylori* compared to PD patients without *H. pylori* infection (Zhong et al., 2022). However, UPDRS-III scores and levodopa doses did not reach statistical significance (Zhong et al., 2022). Furthermore, in a randomized, double-blind, placebo-controlled trial of sixty-seven patients with both PD and *H. pylori*, treating the *H. pylori* infection with triple therapy (amoxicillin, clarithromycin, and omeprazole) had no significant effect on MDS-UPDRS at twelve or fifty-two weeks, and there was no significant effect on quality of life after treatment (Tan et al., 2020). Similarly, one study showed no difference in UPDRS-III after *H. pylori* eradication; however, there was a statistically significant decrease in latency for levodopa to take effect and increase in duration of action of levodopa (Lee et al., 2008). Although several studies, summarized nicely in an article by Camci and colleagues, have demonstrated positive results on motor function and/or levodopa absorption and effect (Camci and Oguz, 2016), the small sample sizes and paucity of randomized controlled trials leaves insufficient evidence to routinely screen for or empirically treat *H. pylori* in patients with PD. However, if the patient is suspected to have a concomitant *H. pylori*-associated disease, treating the *H. pylori* for that indication may confer some benefit for PD symptoms and response to levodopa (Lee et al., 2008).

A second example of the use of antibiotics for gut dysbiosis in PD brings us to the third stop on the gastrointestinal tract tour: the small intestine. In normal conditions, bacterial growth in the small intestine is limited by an acidic, enzyme-rich, and high motility environment. However, in some conditions such as PD where intestinal motility is decreased, bacteria can flourish resulting in small intestinal bacterial overgrowth (SIBO) (Fasano et al., 2013). When SIBO is symptomatic, patients experience bloating, abdominal pain, diarrhea, and/or weight loss, and treatment with a course of antibiotics is generally warranted (Pimentel et al., 2020). In a recent meta-analysis, forty-six percent of patients with PD were found to have SIBO, but the rates of bloating, constipation, and diarrhea were not significantly different between the PD patients with and without SIBO (Li et al., 2021). Thus, it can be unclear if a patient is symptomatic from SIBO warranting treatment with antibiotics or experiencing such GI non-motor symptoms due to other mechanisms in PD.

Whether or not treatment of SIBO with antibiotics helps the GI non-motor symptoms, it could potentially help improve motor fluctuations. A study of PD patients with motor fluctuations and SIBO showed reduced “off” and decreased delayed “on” time (**Table 1**) after eradication of SIBO with rifaximin (Fasano et al., 2013). More recently in an open label study with blinded video evaluations, fourteen patients with PD complicated by dyskinesias or motor fluctuations (**Table 1**) were treated with colon enemas followed by rifaximin for one week and polyethylene glycol-33550 for ten days. Following this treatment all patients exhibited statistically significant improvement in duration and severity of dyskinesias and motor fluctuations as measured by the MDS-UPDRS-IV (motor fluctuation subscale), and sixty-four percent continued to report improvement on the clinical global impressions scale (CGIS) at three-month follow-up (Baizabal-Carvallo et al., 2021). In contrast, Tan et al. did not find a significant difference in motor fluctuations (measured by UPDRS-IV) between SIBO-positive and SIBO-negative patients with PD, but these authors did find significantly worse motor symptoms (measured by UPDRS-III, number of pegs on Pegboard test, and time to complete gait test) in SIBO-positive patients (Tan et al., 2014).

Like eradication of *H. pylori* with antibiotics, there is limited data for the eradication of SIBO with antibiotics in PD. However, antibiotic treatment could be considered for select patients with SIBO and medication and/or deep brain stimulation refractory dyskinesias (Baizabal-Carvallo et al., 2021). Caution and consideration must be taken prior to prescribing antibiotics, however, as antibiotics are also capable of inducing gut dysbiosis by eradicating good gut microbiota from the gastrointestinal tract (Pimentel et al., 2020). The story of antibiotics will continue to unfold to guide our clinical decision-making as current clinical trials are underway to investigate the disease modifying potential of various antibiotics in PD given their role in inflammation and mitochondrial dysfunction, two potential mechanisms in the complex pathogenesis of PD (Yadav et al., 2021).

In addition to antibiotics, caution and consideration should also be taken when prescribing the other key component of triple therapy for *H. pylori*, proton pump inhibitors (PPIs), as regular use has been linked to increased risk of SIBO as well as dementia (Nehra et al., 2018). Additionally, lower gastric acidity has also been shown in mouse models to lower the threshold for infection with prion disease (Martinsen et al., 2009). As we will discuss shortly, “prionoid,” or prion-like propagation of alpha synuclein triggered by some unknown pathogen or toxin in the GI tract is another potential pathogenic mechanism for PD (Braak et al., 2003b; Kodower et al., 2008; Luk et al., 2012; Recasens et al., 2014; Thomzig et al., 2021). Thus, chronic reduction of gastric acidity could render the GI tract and central nervous system more susceptible to such yet unknown pathogens.

### 2.4 Diet

The Mediterranean diet is a diet rich in fresh fruits, vegetables, whole grains, and olive oil and low in saturated fat, sugar, and processed foods. Additionally, the Mediterranean diet is high in fiber which can promote the growth of SCFA-producing bacteria (Bisaglia, 2023), making this diet a possible life-style modification approach to gut dysbiosis. The Mediterranean diet may also target other possible pathogenic mechanisms of PD including neuroinflammation (Gallardo-Fernandez et al., 2020; Almanza-Aguilera et al., 2022) and oxidative stress (Gallardo-Fernandez et al., 2020). Several studies summarized nicely by Bisaglia have shown a decreased risk of PD and prodromal PD with adherence to the Mediterranean diet (Bisaglia, 2023). Research on the effect of the Mediterranean diet on PD symptoms and progression is lacking. A small study of eight patients with PD and eight controls pre and post 5-weeks of Mediterranean diet intervention demonstrated feasibility of adherence to this diet as well as reduced constipation and increased SCFA-producing bacteria after adherence to the Mediterranean diet in the PD group (Rusch et al., 2021). An Iranian study randomized PD patients to either ten weeks of a Mediterranean diet or control diet and found improvement in Montreal Cognitive Assessment (MoCA) total scores as well as executive function, language, attention, concentration, and active memory sub-scores in the Mediterranean diet group (Paknahad et al., 2020). Subsequently, Paknahad and colleagues studied the effect of Mediterranean diet on motor and non-motor symptoms of PD and found significantly improved UPDRS scores in the group randomized to the Mediterranean diet (Paknahad et al., 2022).

A combination of the Mediterranean diet and the Dietary Approach to Stop Hypertension (DASH) diet, also known as the Mediterranean-DASH Intervention for Neurodegenerative Delay (MIND) diet, has also been shown to possibly delay onset of PD, especially in females (Metcalfe-Roach et al., 2021). The MIND diet is similar to the Mediterranean diet but also features low salt, low cholesterol and promotes foods thought to be neuroprotective like blueberries, leafy-green vegetables and oily fish (Knight et al., 2022; Devranis et al., 2023). A cross-sectional study of one thousand two hundred and five patients compared PD severity using the patient-reported outcomes in PD (PRO-PD) score, a thirty-three-question survey of common PD motor and non-motor symptoms, while adhering to the Mediterranean diet versus the MIND diet. The PRO-PD score significantly decreased by 25.6 points per one point increase in Mediterranean diet score and by 52.9 points per point increase in MIND diet score. Thus, while both diets decreased the severity of PD symptoms, the benefit of the MIND diet was more robust which was consistent with prior studies (Fox et al., 2022).

Although more studies are needed to determine the efficacy of the Mediterranean and MIND diets for PD symptoms, the Mediterranean and DASH diet component of the MIND diet are safe interventions already widely recommended to cardiovascular patients (Locke et al., 2018). Thus, keeping the limited current evidence for dietary intervention in PD in mind, if desired, patients with PD could trial the Mediterranean or MIND diet for their symptoms in addition to their existing antiparkinsonian regimen.

### 2.5 Viruses

Bacteria gain the most attention in PD given they are the largest and most well-studied constituents of the gut microbiota (Thursby and Jurge, 2017). Viruses, however, have a long-standing relationship with parkinsonism dating back to the post-encephalitic cases after the Spanish flu in 1918 and continuing over the last three years with SARS-CoV2-associated cases (Smeyne et al., 2021). These cases were caused by cytokine storms that damaged the central nervous system and lead to the onset of parkinsonism during or shortly after the viral infection (Smeyne et al., 2021). This contrasts with idiopathic PD which has a long prodromal period. However, this raises the question of whether an acute viral infection can increase the long-term risk of idiopathic PD. A recent Danish registry study of patients diagnosed with PD between 2000-2016 may have shed some light on this question. This study looked at various viral infections less than five years, more than ten years, and more than fifteen years prior to the diagnosis of PD. Gastrointestinal infections had an odds ratio for PD of 1.38 less than five years from infection but only 1.06 more than ten years from infection. The herpes simplex virus, viral hepatitis, upper respiratory tract infections, and miscellaneous viral infection groups were not significant at any time interval from infection (Cocoros et al., 2021). This contrasts with prior studies that have shown an increased risk of PD with hepatitis C infection (Tsai et al., 2016) and a reduced risk in those who are treated with antivirals compared to untreated patients (Lin et al., 2019; Smeyne et al., 2021). Interestingly, however, the remarkably positive result of this Danish study was that the odds of PD after influenza was more than seventy percent ten years from infection and ninety percent fifteen years from infection (Cocoros et al., 2021).

It is unclear what the mechanism is for increased risk of PD over a decade out from influenza infection. Perhaps remote viral infections can prime the brain to be susceptible to environmental and genetic PD risk factors later in life (Smeyne et al., 2021). An alternative theory is the virus could trigger neuroinflammation and/or propagation of aggregated alpha synuclein from initially exposed tissues such as the gut associated lymphoid tissues in nasopharynx and GI tract to the brain (Jang et al., 2009; Marreiros et al., 2020). This brings us to the last stop on the gastrointestinal tract tour: the gut associated lymphoid tissue.

## 3 Gut-associated lymphoid tissue

The gut associated lymphoid tissue (GALT) includes the tonsils, appendix, Peyer’s patches in the small intestine, and isolated lymphoid follicles along the large intestinal wall. Gut-associated lymphoid tissues sample luminal antigens and contain lymphoid follicles to generate an inflammatory response to certain antigens (Wittig and Zeitz, 2003; Kamada et al., 2013). The appendix is also home to a high density of gut microbiota which can repopulate the intestine following a diarrheal illness (Morbe et al., 2021). Additionally, the appendix is a reservoir of aggregated alpha synuclein in both healthy patients (Gray et al., 2013) and those with PD (Stockholm et al., 2016). It is possible that in certain patients in the right setting of environmental and/or genetic triggers, alpha synuclein could spread from the gut tissues such as the appendix to the brain (Killinger and Labrie, 2019). Thus studies have examined whether eliminating such reservoir with appendectomy could decrease the risk of PD.

Three studies showed decreased risk or delayed onset of PD after appendectomy (Mendes et al., 2015; Killinger et al., 2018; Liu et al., 2020), one study showed no difference in risk of PD (Yilmaz et al., 2017), and two studies showed higher risk of PD following appendectomy (Marras et al., 2016; Svensson et al., 2016). What about tonsillectomy? The tonsils have also been reported to contain aggregated alpha synuclein (Mu et al., 2015). Only two studies have examined the effect of tonsillectomy on the subsequent risk of PD. Both studies showed a non-significant trend toward decreased risk of PD after tonsillectomy (Svensson et al., 2018; Liu et al., 2020). Although resection of these tissues is unlikely to prevent PD through debulking alpha synuclein, the GALT remains of interest as an initial interface with environmental pathogens that enter the body through nasopharyngeal and orogastric routes. For example, in variant Creutzfeldt-Jakob disease, the tonsils and other gut-associated lymphoid tissues are thought to provide a gateway for infectious prions to travel via the peripheral nervous system to the central nervous system (McBride et al., 2001; Hilton, 2005; Svensson et al., 2018). This brings us to our discussion on a possible direct connection between the gut and brain in PD: the vagus nerve.

## 4 A direct connection between the gut and brain: the vagus nerve

In 2003, Heiko Braak, a German neuroanatomist, and his colleagues studied forty-one brains of clinically established PD, sixty-nine brains of incidental PD (no clinical diagnosis but contained the characteristic pathology of PD), and fifty-eight control brains. Braak and colleagues developed a staging system for the pathology of PD such that stage 1 involves PD pathology in the dorsal motor nucleus, olfactory bulb and anterior olfactory nucleus, stage 2 in the pontine tegmentum, stage 3 in the midbrain, stage 4 in the basal forebrain and amygdala, and stages 5 and 6 involve PD pathology more diffusely throughout the neocortex (Braak et al., 2003a). The observation of this predictable, temporal spread of PD pathology led to a further hypothesis for Braak: that some unknown neurotropic pathogen could be the trigger for such staged Lewy body pathology in PD. Braak hypothesized that an unknown pathogen could gain access to the early-affected olfactory structures via the nasopharyngeal route, like some viruses do, and take an anterograde approach to the temporal lobe. That same pathogen could also enter the saliva and make its way to the enteric nervous system. From there, the pathogen could gain access to the early-affected dorsal motor nucleus via retrograde axonal transport along vulnerable, unmyelinated fibers of the vagus nerve (Braak et al., 2003b). This anterograde route from olfactory structures to temporal lobe and retrograde route from the gastrointestinal tract up the vagus nerve to the brain stem became known as the Dual Hit Hypothesis (Hawkes et al., 2007). Animal models have provided evidence that such route of spread from the GI tract and enteric nervous system up the vagus nerve to the medulla is possible (Pan-Montojo et al., 2012; Holmqvist et al., 2014; Kim et al., 2020). For example, one study injected human PD brain lysate containing mono and multimeric forms of alpha synuclein into the intestinal walls of mice and watched using live cell imaging as it was transported via slow and fast axonal transport to the dorsal motor nucleus via the vagus nerve over the course of six days (Holmqvist et al., 2014).

It is important to note that Braak’s hypothesis is criticized for cases that do not follow his staging system (Burke et al., 2008) such as those with Lewy body pathology in the midbrain but not in the lower brainstem (Parkkinen et al., 2004), those with higher stage pathology but no neurological symptoms (Braak et al., 2003a), and those with parkinsonism but no Lewy body pathology at all (Zimprich et al., 2004). In the author’s opinion, given the numerous potential pathogenic mechanisms of PD mentioned earlier (Jankovic and Tan, 2020; Bloem et al., 2021), it is conceivable that the relevant mechanisms at play such as alpha synuclein aggregation and propagation may differ to varying degrees from one patient with PD to the next depending on their specific genetic predispositions and environmental exposures.

### 4.1 Prion-like alpha-synuclein

It is unknown if Braak’s pathogenic trigger is a virus, bacteria, gut dysbiosis, or not a pathogen at all, but rather a toxin such as a pesticide. Whatever the trigger, there has been growing interest in the controversial idea that it could be capable of inducing abnormal aggregation of alpha synuclein which could travel from cell to cell via a prion-like mechanism (Kodower et al., 2008; Luk et al., 2012; Recasens et al., 2014; Thomzig et al., 2021). However, there is debate over whether aggregated alpha synuclein is playing a role in neurodegeneration or is simply a pathological marker of it (Visanji et al., 2013; Killinger and Kordower, 2019; Espay and Okun, 2023). The details of this prion-like mechanism are not fully understood and beyond the scope of this review, but in summary, it is thought that alpha synuclein exists largely as a monomer in neurons, mostly at presynaptic terminals where it is thought to play a role in vesicle transport. Normal monomeric alpha synuclein can undergo post-translational modifications such as phosphorylation that can make it more likely to aggregate into oligomers or fibrils. These aggregated alpha synuclein seeds are then excreted from the cell, possibly in vesicles, and taken up by adjacent cells via multiple potential mechanisms including fusion of membranes or receptor-mediated endocytosis (Jan et al., 2021).

The first evidence for this prion theory came from a study of a patient with PD who underwent transplantation of fetal ventral mesencephalic cells in the bilateral striatum (Kodower et al., 2008). Following this transplant, she had much improvement in her motor symptoms and motor fluctuations. However, eleven years after transplant, her motor symptoms progressed, and she continued to deteriorate until her death from a cardiac arrest. She underwent autopsy, and interestingly, the grafted neurons contained Lewy body-like aggregates of alpha synuclein. This would not be expected of cells that are only fourteen years old and suggested that either there was some pathogenic factor in the brain milieu that continued to affect dopaminergic neurons throughout the disease course, or there was a pathogenic factor that traveled from the patient’s affected cells into the transplanted cells (Kodower et al., 2008).

Subsequent animal studies have suggested such cell to cell spread of alpha synuclein seeds as well. One study inoculated mice brains just above the substantia nigra pars compacta with Lewy body extracts from PD patients (Recasens et al., 2014). By four months the mice exhibited substantia nigra pars compacta dopaminergic cell loss, striatal dopaminergic cell loss, and associated motor coordination and balance issues. They also showed that human alpha synuclein was endocytosed into mice neurons and by four months it was undetectable yet there was pathological murine alpha synuclein accumulation that was resistant to enzymatic digestion. This was not just present in the substantia nigra pars compacta, but in distant sites like striatum and cortical regions. These findings did not occur in cases where inoculations were depleted of alpha synuclein or in alpha synuclein deficient mice. So, the presence of alpha synuclein in this inoculate was key. Similarly, monkeys that underwent either substantia nigra pars compacta or striatal Lewy body injections had, in both cases, a forty percent reduction in striatal dopaminergic neurons and a fifteen percent reduction in substantia nigra pars compacta dopaminergic neurons. There was also spread of alpha synuclein pathology to distant brain regions (Recasens et al., 2014). Another animal study showed that even injections of non-CNS alpha synuclein, from the stomach wall of a patient with PD, into the brains of mice can cause pathological endogenous alpha synuclein seeding (Thomzig et al., 2021). This did not happen when injected with a PD patient’s blood or muscle tissue, where pathological alpha synuclein is not found. This again suggests the presence of alpha synuclein in the inoculate was key. The incubation time of about five hundred days post injection in that study enabled all animals to demonstrate the pathological alpha synuclein that was not detected even four hundred days post injection. This was a potential weakness of a negative alpha synuclein seeding study using the same mouse model that examined brains only three hundred sixty days post-injection (Thomzig et al., 2021).

### 4.2 Vagotomy

Further suggestion of a direct brain-gut connection via the vagus nerve comes from studies of vagotomy. One study administered rotenone to mice and observed initiation of alpha synuclein accumulation in the enteric nervous system that later spread to the dorsal motor nucleus and substantia nigra. Resection of the vagus nerve before rotenone administration was able to prevent that pattern of alpha synuclein spread and associated dopaminergic cell death in the substantia nigra (Pan-Montojo et al., 2012). Similarly, another study injected alpha synuclein pre-formed fibrils into the duodenum of mice and observed alpha synuclein in the dorsal motor nucleus one month after injection, the amygdala three months after injection, and in the striatum and prefrontal cortex seven to ten months after injection, corresponding to Braak’s caudal to rostral progression (Kim et al., 2020). The mice also exhibited motor and non-motor symptoms as the pathology spread. Truncal vagotomy prior to injection of the alpha synuclein pre-formed fibrils prevented the spread, dopaminergic cell loss in the substantia nigra pars compacta, and the motor and non-motor symptoms observed in the mice in this study (Kim et al., 2020).

In humans, one study found decreased risk of PD more than five years after a truncal vagotomy (Liu et al., 2017), one study found a non-significant trend toward reduced risk more than five years after a truncal vagotomy (Svensson et al., 2015), and one study found no change in risk after truncal vagotomy (Tysnes et al., 2015). These were registry-based studies of patients who underwent vagotomy for other issues, such as refractory peptic ulcer disease, that involve inflammation of the GI tract. GI inflammation, as we will shortly discuss further, can lead to activation of the immune system that can influence the brain. Thus, removing the direct route to the brain via vagotomy does not eliminate the indirect path of immune system activation, and in some cases, could facilitate that inflammatory path. Surgery itself transiently increases inflammation, but beyond this, vagotomy can lead to complications like diarrhea and nutritional disturbances (Skellenger and Jordan, 1983) that could promote a dysbiotic and/or inflammatory gut environment.

## 5 An indirect connection between the gut and brain: the immune system

We have just discussed a direct connection between the gut and the brain via the vagus nerve, but indirect connections between the gut and brain exist as well: via the endocrine and immune systems. There is evidence for an endocrine connection between the gastrointestinal tract and the brain summarized nicely in a review by Holzer et al. (Holzer and Farzi, 2014). While gut microbiota can synthesize neuropeptides and hormones to influence the brain via such endocrine connection, we will focus on the immune connection between the gut and the brain as it is overall more relevant to our discussion of microbes throughout the gastrointestinal tract. Additionally, in some cases endocrine influences, such as glucagon-like peptide 1 (GLP-1), work on microglia and downstream inflammatory pathways (Manfready et al., 2022).

In the section on the gut microbiota we discussed that SCFA-producing bacteria maintain gut mucosal barrier integrity and lack of such bacteria in PD may lead to a leaky gut barrier (Thursby and Jurge, 2017; Metta et al., 2022). Additionally, SCFA are also reported to maintain the blood brain and blood cerebrospinal fluid barriers (Hoyles et al., 2018; Liu et al., 2021; Xie et al., 2023), so lack of SCFA-producing bacteria in PD may leave the brain more vulnerable to inflammatory cells, pathogens, and toxins (Mirzaei et al., 2021).

The leaky gut barrier in PD may enable exposure to the gram-negative bacterial antigen, lipopolysaccharide (LPS) which has been implicated in neuroinflammation and neurodegeneration (Qin et al., 2007; Valenzuela-Arzeta et al., 2023). The mechanism for this is unclear but may be mediated by toll-like receptor 4 (TLR4) (Chakravarty and Herkenham, 2005; Perez-Pardo et al., 2019; Zhao et al., 2021). Perez-Pardo et al. took sigmoid colonic mucosal biopsies from patients with and without PD and found decreased SCFA-producing bacteria, increased TLR4 and CD3+ T cell mRNA expression, increased TLR4+ cells and CD3+ T cells, and increased TLR4 downstream signaling products (proinflammatory cytokines) in the lamina propria of PD patients compared to healthy controls. Other TLRs were not upregulated. These authors found the same result in mice given rotenone. However, when they exposed TLR4-/- mice to rotenone, the TLR4-/- mice had less CD3+ T cells in their intestinal mucosa, less microglia activation in their substantial nigra, less dopaminergic cell less in their substantia nigra, performed better motorically, and had better intestinal barrier integrity and faster colonic transit time than the wild type mice exposed to rotenone (Perez-Pardo et al., 2019).

The latter study suggests that activation of TL4Rs in the gut leads to release of proinflammatory cytokines that can lead to neuroinflammation and neurodegeneration. Indeed, patients with PD have been found to have elevated levels of various pro-inflammatory cytokines in the blood (Nagatsu et al., 2000; Heidari et al., 2022) which could activate microglia in the substantia nigra and other brain regions which then release more pro-inflammatory molecules to attract peripheral inflammatory cells like monocytes, T and B lymphocytes into the brain (Heidari et al., 2022). Alternatively, bacterial translocation through a leaky PD gut into the blood and through a leaky blood brain barrier into the brain could expose microglial TLR4s to LPS directly to induce neuroinflammation and neurodegeneration in the brain (Baizabal-Carvallo and Alonso-Juarez, 2020).

In addition to binding LPS, TLR4s on microglia have been found to recognize oligomeric alpha-synuclein as well, resulting in up to one hundred times greater TNF-alpha production in wild type mice than TLR4 -/- mice (Hughes et al., 2019; Heidari et al., 2022). This represents another example of how the direct and indirect brain-gut connection pathways overlap: aggregated alpha synuclein entering the brain via the vagus nerve direct pathway may stimulate TLR4-induced neuroinflammation which may already be ongoing via the indirect systemic immune system pathway discussed above.

## 6 Conclusions

On our tour through the gastrointestinal tract, we have discussed how gut dysbiosis in the large intestine, *H. pylori* in the stomach, bacterial overgrowth in the small intestine, and pathogens and proinoid alpha synuclein in the GALT can potentially influence the symptoms and/or progression of PD. These GI-related triggers could take a direct route from the gut to the brain via the vagus nerve or indirectly influence the brain via the immune system. However, we have seen that these pathways are interconnected contributing to the complexity of the pathogenesis of PD, and this complexity is only in relation to one organ system: the gastrointestinal system!

With so many potential pathophysiological mechanisms for PD it is difficult to conclude the degree to which any of these gut factors play a role in the etiology, progression, or severity of disease in any one individual patient with PD. Nevertheless, the gut factors discussed in this review have become “hot topics” of interest that have and will continue to form the basis for experimental therapeutic interventions for PD.

Probiotics (other than for constipation), antibiotics, and fecal microbiota transplant will all require further study to determine their safety and efficacy for patients with PD. Dietary interventions such as the Mediterranean and MIND diets also require further study but are relatively safe lifestyle modifications that can be trialed by patients if desired. Antivirals like amantadine are currently used for the symptomatic treatment of PD, and amantadine has shown some evidence for delaying levodopa-induced dyskinesias when started early (Wang et al., 2022). Finally, three drugs targeting inflammation, eight drugs targeting the microbiome/GI tract, and fourteen drugs targeting alpha-synuclein are currently in the pipeline as of January 2023 (McFarthing et al., 2023). Overall, there is high interest in the role of the gastrointestinal tract in PD, but much work remains to understand its relevance and utility for patients with Parkinson’s disease.

## Author contributions

CK and AH contributed to the design and implementation of the review, AH wrote the first draft, and CK, AH contributed to the final writing of the manuscript. All authors contributed to the article and approved the submitted version.
